# Transcranial Electrical Stimulation Accelerates Human Sleep Homeostasis

**DOI:** 10.1371/journal.pcbi.1002898

**Published:** 2013-02-14

**Authors:** Davide Reato, Fernando Gasca, Abhishek Datta, Marom Bikson, Lisa Marshall, Lucas C. Parra

**Affiliations:** 1 Department of Biomedical Engineering, The City College of the City University of New York, New York, New York, United States of America; 2 Institute for Robotics and Cognitive Systems, University of Lübeck, Lübeck, Germany; 3 Graduate School for Computing in Medicine and Life Sciences, University of Lübeck, Lübeck, Germany; 4 Department of Neuroendocrinology, University of Lübeck, Lübeck, Germany; Research Center Jülich, Germany

## Abstract

The sleeping brain exhibits characteristic slow-wave activity which decays over the course of the night. This decay is thought to result from homeostatic synaptic downscaling. Transcranial electrical stimulation can entrain slow-wave oscillations (SWO) in the human electro-encephalogram (EEG). A computational model of the underlying mechanism predicts that firing rates are predominantly increased during stimulation. Assuming that synaptic homeostasis is driven by average firing rates, we expected an acceleration of synaptic downscaling *during* stimulation, which is compensated by a reduced drive *after* stimulation. We show that 25 minutes of transcranial electrical stimulation, as predicted, reduced the decay of SWO in the remainder of the night. Anatomically accurate simulations of the field intensities on human cortex precisely matched the effect size in different EEG electrodes. Together these results suggest a mechanistic link between electrical stimulation and accelerated synaptic homeostasis in human sleep.

## Author Summary

Sleep pressure is reflected in the power of slow-wave activity: it is high after extended wakefulness and gradually decays in the course of the night. Transcranial stimulation with slow-oscillating currents can entrain electro-encephalographic slow-wave oscillations (SWO) and transiently increase their power. Motivated by the results from a multi-scale computational model, we tested in humans whether 25 minutes of transcranial stimulation attenuates the decay of SWO in the remainder of the night. A Finite-Element Model (FEM) is used to estimate the current flow in the brain and a network model of spiking neurons determines the resultant effect on SWO. This multi-scale model predicted increased neuronal firing rates leading to accelerated synaptic downscaling. As a consequence, the decay of SWO power and spatial coherence after stimulation is reduced. In addition to reduced decay rate, the model was also able to successfully predict, in the human experiments, the spatial distribution of the effect across EEG electrodes. These combined experimental and modeling results suggest a mechanism by which electrical stimulation can accelerate synaptic homeostasis and thereby influence a putative process of sleep regulation. The ability to accelerate the homeostatic function of sleep may have important practical implications.

## Introduction

Human sleep is characterized by distinct sleep stages which can be readily identified in the electroencephalogram (EEG). Of particular interest is the activity in the 0.5–4 Hz frequency band known as slow-wave activity (SWA). The power of SWA increases following extended waking and decreases in power and spatial coherence throughout the night [1], [2]. SWA activity is thought to reflect a homeostatic mechanism that regulates sleep [3]. These changes in power have been hypothesized to result from potentiation and downscaling of synaptic connections during wakefulness and sleep respectively [4]–[9].

Homeostatic plasticity refers to a physiological feedback mechanism that regulates average firing rates by altering synaptic strength: high firing rates lead to synaptic depression and low firing rates to potentiation [10]. A link between homeostatic plasticity and sleep homeostasis is supported by the parallels between firing rates and SWA: namely, extended waking results in increased cortical firing rates at the beginning of sleep, and firing rate decays again during sleep [11].

Here we consider slow-wave oscillations (SWO, 0.5–1 Hz) in the human EEG as a marker for sleep homeostasis and its modulation by transcranial electrical stimulation. We found that a relatively short 25 minutes of stimulation in humans during slow-wave sleep at the beginning of the night had a lasting effect on homeostatic decay of SWO in the hours following stimulation.

The effects of transcranial electrical stimulation on brain activity have been the subject of intense investigation in the last decade [12], [13]. A number of studies show specific enhancement in human cognitive performance including memory, language, computational, and executive function [14]–[17]. The mechanisms leading to the observed cognitive effects of weak electrical stimulation in human behavioral studies remain fundamentally unaddressed. The current mechanistic explanation is limited to the notion of neuronal excitability where function is “increased” or “decreased” by virtue of neuronal polarization with anodal or cathodal stimulation respectively. However, the basic physics of current flow calls this simple notion into question as cortical folding leads to varying polarity across cortex making the origins of polarity specific effects unclear [18]. Furthermore, while acute effects of uniform week electric fields are well characterized, including modulation of firing rates [19], it is less clear how these acute effects translate into specific long term effects.

We hypothesized that stimulation during slow-wave sleep alters neuronal firing rates, which would modulate homeostatic synaptic downscaling and thus alter the homeostatic decay of SWO. A multi-scale computational model makes this hypothesis explicit by linking the macroscopic domains of current flow in the entire head with the microscopic cellular effects of polarization. The model shows that network dynamics of SWA can rectify bi-directional polarization leading to an unidirectional increase of firing rates and synaptic downscaling. A number of predicted effects of stimulation on SWO are subsequently confirmed by the present human EEG sleep data. Specifically, the data confirmed the prediction of diminished SWO decay in the hours after stimulation, and the multi-scale model accurately predicted the effect sizes across multiple scalp electrodes.

The ability to accelerate sleep homeostasis may have important practical implications given that SWA is widely considered to be a marker of the restorative power of sleep.

## Results

### SWO power and spatial coherence decay with time during sleep

**Figure 1:**
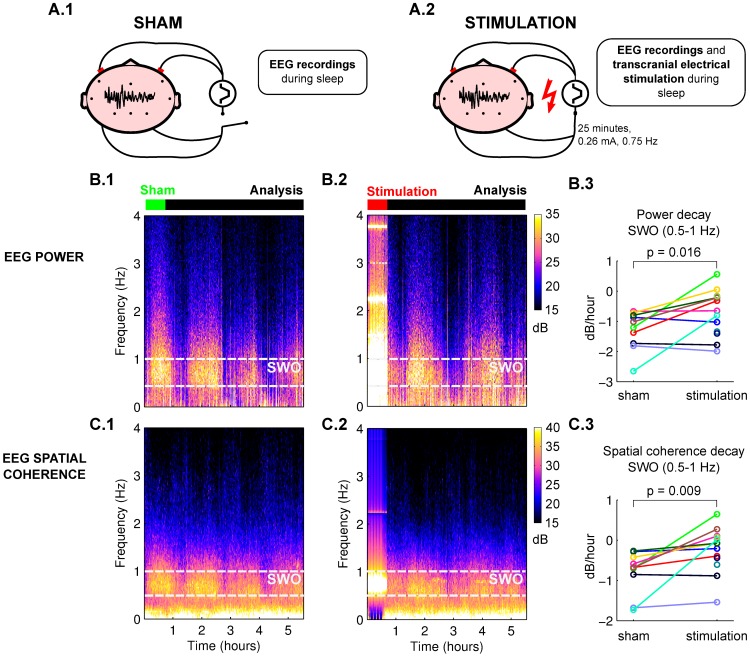
Transcranial electrical stimulation affects power and spatial coherence of human EEG during sleep. EEG is recorded from 11 electrode locations, stimulation electrodes are placed bilaterally on the scalp. **A.1**: In the sham condition stimulation electrodes were placed but no current was applied. **A.2**: In the stimulation condition slow-oscillating (0.75 Hz) current is applied for 25 minutes at the beginning of the night. **B.1–B.2**: Spectrograms of power in sham and stimulation conditions during the night in the human EEG data (average across subjects, Pz electrode). **C.1–C.2**: Spectrograms of spatial coherence between Pz and other EEG electrodes. **B.3**: Decay rate of power in the SWO band during the analysis period (4.5 hours after the stimulation). Colors indicate subject. **C.3**: Decay rate for spatial coherence in the SWO band in the analysis period. Stimulation and sham condition differ significantly in decay rate for both power and coherence (paired shuffled statistics, N = 10 subjects).

In a study on memory consolidation during sleep [14] Marshall et al. stimulated participants during the first period of slow-wave sleep with slow-oscillating unipolar stimulation (0.26 mA switched on and off at 0.75 Hz). Positive (anodal) electrodes were placed bilaterally over lateral prefrontal cortex and negative (cathodal) electrodes over left and right mastoids. EEG was recorded simultaneously from 11 electrodes (Figures 1.A.1, 1.A.2). To characterize the long term effects of stimulation on slow-wave activity, we computed here for each participant the power-spectrum over the course of the night. Slow-wave activity (0.5 Hz–4 Hz) is modulated in time as participants cycle through non-REM and REM sleep stages (Figure 1.B.1, average over 10 participants). Note that the EEG data were aligned based on sleep stages (see Materials and Methods), and sleep-stage cycle-durations are fairly reproducible across subjects [14], [20]. We estimated decay rates of power and coherence as a linear fit on a logarithmic scale (dB), which corresponds to an exponential decay in time (example traces in Figure S1.A–B) [21]–[23]. In the present data the homeostatic decay of power in the band of slow-wave oscillations (0.5 Hz–1 Hz) amounted to −1.22
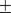
0.18 dB/hour (mean 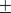 sem, p-value = 0.0001, N = 10, Student's t-test, Figure 1.B.3, analysis window of 4.5 h marked in black, see Materials and Methods). In addition to changes in power, the computational model, which will be presented in the following sections, predicted that the spatial coherence of SWO should also decay. The coherence-spectrum between electrode pairs was computed and averaged across all pairs (Figure 1.C.1, average over 10 participants). In the band of SWO, coherence decays at a rate of −0.70
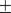
0.12 dB/hour (mean 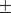 sem, p-value 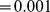, N = 10, Student's t-test, Figure 1.C.3). The present measure of spatial coherence is normalized by power. Thus, its decay does not simply capture a decrease in power but reflects instead a break-up of large scale coherent oscillations over distant cortical areas consistent with recent recordings in humans [2].

### Homeostatic decay of SWO is altered by slow-oscillating transcranial electrical stimulation

Our hypothesis on homeostatic plasticity predicted that the decay of SWO should be altered by the transcranial slow-oscillating stimulation administered to participants for 25 minutes (spectrograms in Figures 1.B.2, 1.C.2). Specifically, we expected a reduced rate of decay in both power and spatial coherence in the hours following stimulation. This prediction was confirmed by the present data: the post-stimulation decay rate for power averaged over all electrodes is reduced to −0.69
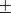
0.18 dB/hour (N = 10, paired shuffled statistics, p = 0.016, Figure 1.B.3) and similarly, the rate of spatial coherence is reduced to −0.15
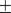
0.12 dB/hour (N = 10, p = 0.009, Figure 1.C.3). Significant differences in decay rate are found also when analyzing individual electrodes in isolation (p-values corrected for false discovery rate are between 0.013 and 0.035 for all electrodes except F7 with p = 0.132) and the same is true for coherence (p-values between 0.013 and 0.031 except T3 with p = 0.063). The wider band of SWA (0.5–4 Hz) yielded essentially the same results (p
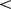
0.05). Changes in sleep structure are hard to assess from the average spectrogram in Figures 1.B-0.C. Previous analysis already dismissed possible changes in terms of time spent in different sleep stages during the 60 minutes after the stimulation or the whole night, nor were there differences in the number of sleep cycles [14].

In summary, as predicted, the decay of SWA, which is widely considered to be a marker of sleep homeostasis, is reduced in the hours following electrical stimulation. In the following section we make quantitative predictions of this phenomenon by detailing our hypothesis in the form of a multi-scale computational model. We include a finite-element model of the current flow in the brain as well as a network model for slow wave oscillations.

### Transcranial electrical stimulation in humans polarizes the cortical surface with mixed polarity

**Figure 2:**
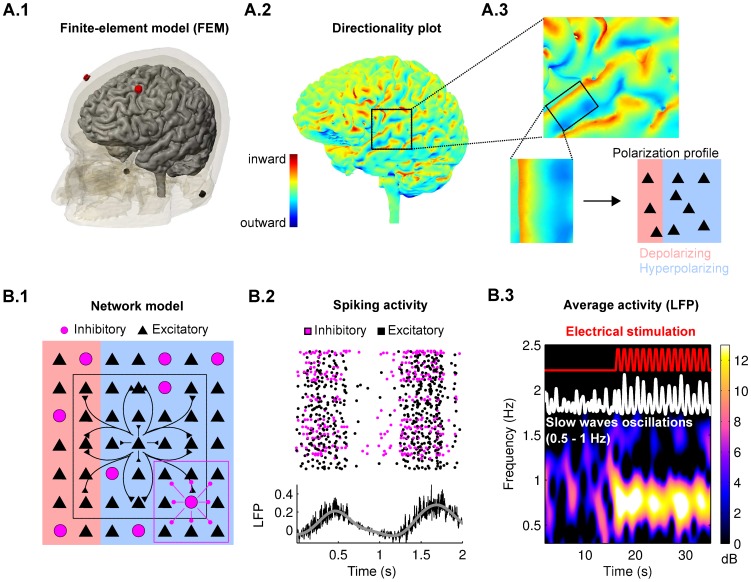
Multi-scale model of transcranial electrical stimulation. **A.1**: A Finite-Element Model (FEM) of tissue resistance was used to calculate electric field magnitude and direction at the cortical level in response to currents applied on the scalp. Electrodes were placed at the same locations as in the human sleep experiments (red = anodal = inward, blue = cathodal = outward). **A.2**: Applied electric fields show mixed polarities throughout cortex due to cortical folding (color indicates radial field component orthogonal to cortical surface). **A.3**: Cortical neurons in close proximity may be exposed to depolarizing (inward) and hyperpolarizing (outward) radial fields. **B.1**: The network model consists of excitatory and inhibitory neurons arranged in a 2D-lattice with long- and short-range synaptic connections respectively. Field magnitude and polarity of the stimulation applied follow the FEM computations and were applied to the network depending on the location within the lattice (applied polarity indicated with red/blue shading). **B.2**: Spiking activity of neurons in the network reproduces the typical UP and DOWN states of SWO. The LFP (black line) is determined as the average of the post-synaptic currents (gray line, LFP low-pass filtered, cut-off frequency 2.5 Hz). **B.3**: Example of network activity (LFP in white). Oscillation is in the range of human SWO (0.5–1 Hz). Spectrogram indicates power of this signal. Red curve indicates slow-oscillating ON/OFF stimulation (0.75 Hz) which is applied to the excitatory neurons in the network.

To determine the expected effects of stimulation for this specific human experiment we first simulated the current flow in an anatomically accurate model of the head (Figure 2.A.1, see Materials and Methods). Electrodes were placed as in the human experiments and currents were monophasic (ON/OFF). As a result of the typical folding of human cortex, different cortical regions experience electric fields of varying magnitudes and, more importantly, of opposing polarities (blue and red in Figure 2.A.2). Thus, neurons in adjacent cortical areas will experience opposing membrane polarizations (Figure 2.A.3). This finding is not unique to the specific electrode montage [18].

### Slow-oscillating stimulation increases firing rate during SWO despite mixed polarity

To examine the effect of differing stimulation polarities on SWO we developed a simple network model of UP/DOWN state transitions. Single-compartment excitatory and inhibitory spiking neurons were recursively connected and arranged on a 2D lattice (900 neurons, Figure 2.B.1). The model reproduces slow-wave oscillations by virtue of an activity-dependent slow recovery variable in a fashion comparable to previous models of SWO [9], [24]–[26] (Figure 2.B.2). The recovery variable acts to decrease neuronal excitability after periods of high activity (UP-state) and recovers after periods of quiescence (DOWN-state). The parameters of the model were chosen to reproduce key features of SWO in humans, such as oscillation frequency and coherence time, and the firing rate of single neurons was adjusted to match animal *in vitro* data (Figure 2.B.3, see Materials and Methods). Note that network parameters were chosen here to reproduce the irregular slow-wave pattern typical of human EEG data (i.e. short coherence times, see Materials and Methods). These contrast the very regular oscillations often measured in *in-vitro* preparations [26], [27] which can be readily reproduced by the present model by increasing the strength of synaptic connections (see Materials and Methods). The effects of weak-field stimulation were implemented as a weak current injection to pyramidal neurons. The specific model of field-to-neuron coupling was validated at multiple frequencies in terms of firing rates, spike timing and entrainment using rat hippocampal slice recordings [19]. The same modeling approach was also used to model acute entrainment of slow waves oscillations in cortical ferret slices [28].

**Figure 3:**
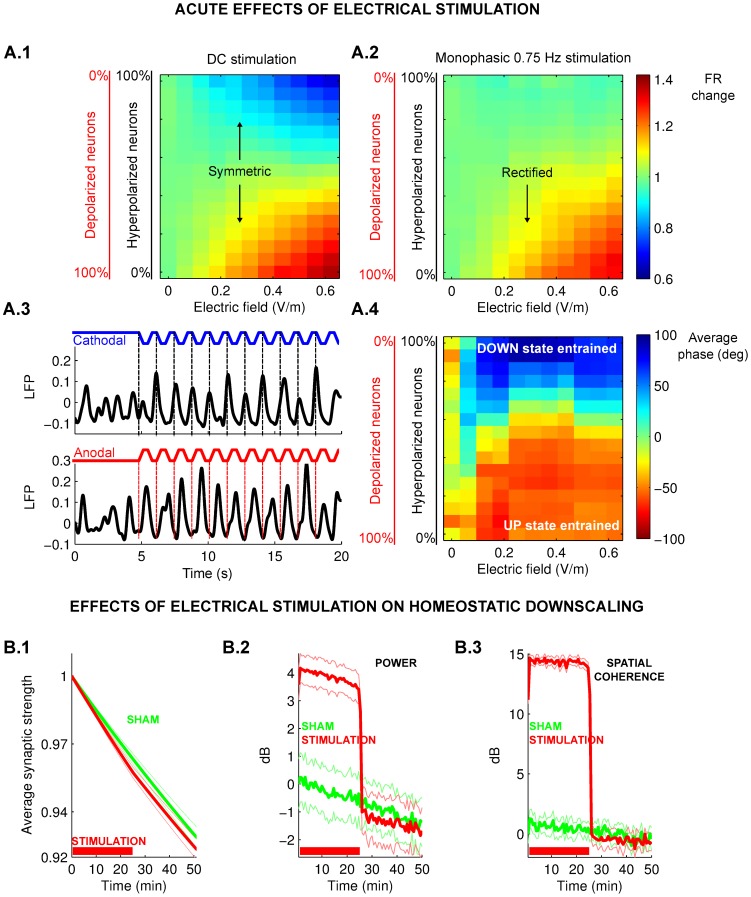
Entrainment of network oscillations to weak electric field stimulation and effects on homeostatic synaptic downscaling. **A.1**: Change in average firing rate by constant current stimulation (DC) as a function of the stimulation intensity and the fraction of neurons polarized in either direction (depolarized and hyper-polarized). Firing rate increases or decreases depending on predominant polarity of field stimulation. **A.2**: Change in average firing rate during slow ON/OFF stimulation (0.75 Hz) as in A.1. Note the rectification of the effect of fields on firing rate, which now only increases for inward stimulation but does not decrease for outward currents. **A.3**: Entrainment of the network with 0.31 V/m monophasic ON/OFF stimulation for purely cathodal (blue) or purely anodal (red) field. Note that the ON period of stimulation aligns with the DOWN state for cathodal and with the UP state for anodal stimulation. **A.4**: Phase of network oscillations relative to the oscillating stimulus as a function of the same parameters as in A.1. **B.1**: Applying a firing-rate dependent synaptic update rule leads to a gradual decrease of average synaptic strength given the relatively high firing rate of the UP state. Electrical stimulation (red curve) accelerates this effect relative to sham (green curve). **B.2–B.3**: The immediate effect of decreased synaptic connections is a decrease in power and spatial coherence of network oscillations. In the stimulation condition, both power and spatial coherence after the stimulation are lower than in sham condition and they decay at a slower rate after stimulation. The results represent 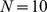 simulations with randomly chosen synaptic connections, bars indicate standard error of the mean.

Different areas of the network were subjected to depolarizing or hyperpolarizing fields corresponding to the mixed polarities of the macroscopic field distributions (Figure 2.B.1). We find that when the network is subjected to constant current stimulation, average firing rates during slow-wave oscillations were increased or decreased depending on the predominant stimulation polarity (Figure 3.A.1). However, when stimulation was turned on and off at the same rate as the slow-oscillations (0.75 Hz), firing rate was only increased (Figure 3.A.2). This remarkable rectification of field-effects on firing rate is the result of the entrainment of the slow-wave oscillation to the applied oscillating field as will be explained below.

### Entrainment of SWO to oscillating stimulation explains rectification of firing rate effect

The network model suggests that weak oscillating stimulation can entrain SWO even for very low amplitude fields (Figure S2.A) and that entrainment results from a modulation of the duration of the UP and DOWN state (Figures S2.B.1-S2.B.2). Entrainment, as previously reported [14] is confirmed here with the present analysis of EEG data (Figure S2.C.1-C.2, Pz electrode, Rayleigh test, 5 trials per 13 subjects considered, p = 0.017). Entrainment of UP/DOWN-state transitions for weak applied fields have also been reported in ferret slices [28] and spiking activity was also entrained in *in vivo* recordings in rat [29]. Neither study reported any long term effects of fields on SWO.

For monophasic stimulation, as in the present study, entrainment occurs regardless of polarity, but does so with opposing phase for opposing polarities (Figure 3.A.3). In the case of depolarizing stimulation (anodal with currents flowing into cortex), the ON period of stimulation aligns with the UP-state, while in the case of hyperpolarizing stimulation (cathodal with currents flowing out of cortex), the ON period aligns with the DOWN-state (Figure 3.A.4). The depolarizing field during the UP-state can increase the firing rate of this active state. However, hyperpolarizing fields during the DOWN-state can not reduce firing rate as the network is already quiescent.

Thus, while DC stimulation may lead to mixed effects on firing rate across space, applying slow-oscillating ON/OFF stimulation during SWO may rectify the effects of fields leading to an unidirectional increase in firing rate.

### Electrical stimulation affects homeostatic downscaling in the network model

*In vivo* animal experiments suggest that synapses undergo downscaling during sleep [5] and that this coincides with a reduction in firing rates [11]. This is consistent with homeostatic synaptic plasticity, which adapts synaptic strength so as to stabilize firing rate to a set level [30]. We implemented here a slow, activity-dependent negative feedback on excitatory synaptic strength. Given the relatively high firing rate of the UP-state, this leads to widespread synaptic downscaling (green curve in Figure 3.B.1), and in turn, to a decrease in the power of slow-wave oscillations in the course of time (Figure 3.B.2). Spatial coherence of slow-wave oscillations also decreased with time (Figure 3.B.3). Both results are consistent in direction and magnitude with the present human EEG data (Figures 1.B.1 and 1.C.1).

**Figure 4:**
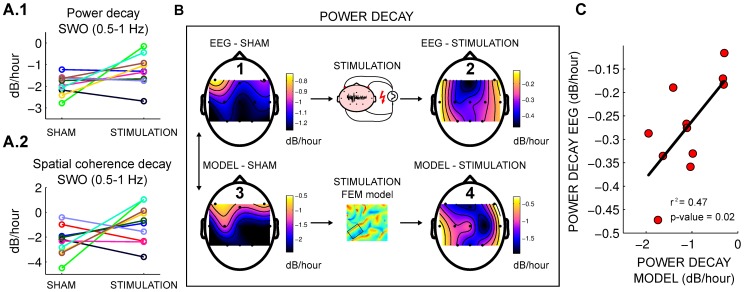
Multi-scale model predicted the after-effects of the stimulation and their variation across electrode locations. **A.1–A.2**: Decay rate after the stimulation for sham and stimulation condition in the computational model for power and spatial coherence. Compare this to the measurements in the human EEG data in Figure 1.B.3–C.3. **B**: Spatial distribution of decay rates across the 11 scalp electrodes averaged across subjects. **1**: for EEG power in sham condition, **2**: for EEG power in stimulation condition, **3**: Approximate fit of network model parameters to match human sham data for each electrode location, **4**: Resulting decay rates for the location-matched network models with stimulation intensity and polarity determined from the FEM model in a 1 cm vicinity of each electrode location. **C**: Change in decay rate of power for stimulation condition. Each point represents one electrode with EEG data on vertical and multi-scale model on horizontal axis. EEG data significantly correlates with model prediction.

We argued above that slow-oscillating stimulation leads to an acute increase of firing rate, even at the small field intensities expected on human cortex of less than 0.5 V/m. In the network model this increased firing rate caused faster synaptic downscaling (Figure 3.B.1, using a field magnitude of 0.31 V/m). With this accelerated downscaling *during* stimulation, at the end of stimulation, firing rates are reduced as compared to the sham condition. Thus, with a diminished drive for downscaling, in the hours *after* stimulation the rate of SWO decay was correspondingly reduced – in power as well as spatial coherence (decays in Figures 3.B.2–3.B.3 and results in Figures 4.A.1–4.A.2).

In the human experiment acceleration *during* stimulation could not be measured directly because entrainment and stimulation artifact distort the endogenous EEG signal. Instead, we measured the slope of decay *after* stimulation (Figures 1.B.3 and 1.C.3). These measures matched the model predictions shown in Figures 4.A.1–4.A.2: the difference in the decay for power between the stimulation and sham conditions in the EEG data is 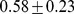 dB/hour and 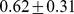 dB/hour in the computational model; for spatial coherence the difference in decay rate is 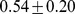 dB/hour and 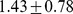 dB/hour respectively.

### Accurate spatial prediction of effect size

To further test the link between stimulation and downscaling, we analyzed the effect size for each of the 11 recording sites. For the human experiment the rate of decay in power was determined for each electrode and averaged across subjects for the sham and stimulation conditions (Figure 4.B.1–4.B.2). We ran the model without stimulation using random synaptic weights and selected for each location a set of weights that approximately matched spatially the EEG sham condition in terms of their decay rate (Figure 4.B.3). We then applied stimulation to the model of each “location” using the intensity distribution of fields found in the FEM model in the vicinity of each electrode. We used the field intensity orthogonal to the cortical surface since cell polarization is approximately proportional to the field intensity in the main axis of pyramidal cells [31]. The average value of the electric field chosen was 0.93 V/m (in this case the stimulation is depolarizing or hyperpolarizing for different locations of the network; see Materials and Methods). This resulted in a decay rate for each “location” as shown in Figure 4.B.4. The spatial distribution is remarkably similar to the one observed in the human EEG. Indeed, the effect size of stimulation versus sham across electrodes was significantly correlated with the predicted values (N = 11 electrodes, 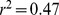, p = 0.02, Figure 4.C).

In summary, the model not only explained the systematic reduction in decay rate of SWO power after stimulation despite mixed polarity stimulation, but it also predicted the effect size in each location by considering the specific mix of polarities near each electrode.

## Discussion

Slow-wave activity has long been associated with the restorative function of sleep [32] and recovery from wakefulness [5], [21]. EEG slow-wave oscillations reflect periodic transitions between UP and DOWN states broadly distributed over the cortex [33] and are thought to be involved in plastic mechanisms [34]. The power of SWA has been linked to learning; for instance, practice on a visuomotor task preceding sleep increases SWA and its strength correlates with task performance following sleep [6], [8]. SWA is also hypothesized to play a crucial role in memory consolidation by virtue of its ability to group the activity of various brain rhythms [35] (e.g. hippocampal ripples; [36], [37] and thalamo-cortical spindles [38].)

A predominant feature of SWA is its decay in the course of the night. Many investigators attribute this decay to homeostatic downscaling of synaptic strength [5], [6], [9]. In their view, synaptic connections that became stronger during wakefulness are reduced in magnitude during sleep. Consistent with homeostatic synaptic plasticity, this decrease coincides with a reduction in firing rates [11]. Homeostatic plasticity represents a negative feedback that adapts synaptic strength resulting in a steady level of neuronal activity [10]. Synaptic downscaling during sleep has been postulated to serve a number of important functions, such as maintaining computational efficiency of the brain by increasing the signal-to-noise ratio of synaptically decoded information [35]; allowing maximum storage efficiency while preventing hyperactivity [39]; and maintaining synaptic normalization [40]. The physiological substrate for the scaling of synaptic connections could be explained by considering that the levels of neuromodulators strongly differ from waking to NREM sleep, for example the concentrations of acetylcholine [41], [42] and norepinephrine [43] are significantly altered. Alternatively, spike-timing dependent plasticity (STDP) during neuronal bursts in slow-wave sleep may favor synaptic depression [44]. Downscaling has also been proposed to results from bursts of activity leading to long-term-depression during NREM sleep [45]. Recent studies also point to a possible role of glial cells in determining synaptic scaling. [46].

We previously showed that slow-oscillating transcranial electrical stimulation can modify endogenous slow oscillatory activity on a short term basis [14]. The question for the present work was whether cortical homeostatic mechanisms are influenced by slowly oscillating transcranial stimulation.

Anatomically accurate models of current-flow in transcranial stimulation estimate that the electric fields induced at the cortical level for a typical 2 mA stimulation are at most 1 V/m [18]. This may polarize a cell by no more than a fraction of a millivolt [31], [47]. While these intensities seem very small, there are a number of *in vitro* and *in vivo* experiments explaining the basic mechanisms by which such low-amplitude electric fields may nevertheless acutely alter neuronal activity, both at the single cell [48] and at the network level [19], [49]–[51]. In particular, it has already been shown, both experimentally and using computational models [19], [28], that the effects resulting from the modest membrane polarization of isolated neurons are significantly amplified on the network level due to the dynamic nature of network activity. This can result in altered firing rates and altered oscillatory rhythms. For instance, the modulation of gamma activity with theta oscillations in the hippocampus is conceivably entirely due to the small fields generated endogenously in the theta band [19]. Similarly, slow-wave activity can be entrained by very weak endogenous fields *in vitro* [28] or weak applied currents *in vivo* [29]. Most importantly, however, there are a multitude of studies in human showing long term plastic effects (e.g. [13], [52]–[56], just to name a few). These are often simply described as lasting changes in neuronal excitability [57]. However, the mechanisms by which weak stimulation could modulate/induce plasticity are less well understood. In humans, both enhancing and suppressing effects have been found with either polarity of stimulation. Some studies argue that depolarizing currents enhance glutamatergic or NMDA dependent Hebbian-type plasticity [58], [59], while other studies have invoked homeostatic plasticity [60]. Lasting effects on synaptic efficacy have only recently been found *in vitro* [61], [62]. These studies demonstrate that very specific conditions on network activity are required in addition to weak-field stimulation in order to observe lasting changes in synaptic efficacy [63].

In the present study we have aimed to provide a detailed explanation of how weak fields, which are capable of modulating network firing rates [19], may alter ongoing homeostatic plasticity, and how this translates into observable macroscopic effects on EEG slow-wave oscillations. Crucial for our predictions was a network model of slow-wave oscillations that is based on UP/DOWN state transitions. We showed that SWO entrain to weak-field slow-oscillatory stimulation consistent with experiments *in vitro* [28] and *in vivo* [29]. We also confirmed entrainment here again on the human EEG data (Figure S2.C.1). The model exhibited entrainment for depolarizing, hyperpolarizing and mixed polarity stimulation (Figures 3.A.3–3.A.4). Importantly, we demonstrate how this entrainment rectifies the effects of fields of mixed polarity to result only in increased firing rates (Figure 3.A.2). When combined with homeostatic plasticity, the model reproduced slow-wave decay in power similarly to previous more complex computational models [9] (Figure 3.B.2). Interestingly, the present model also reproduced the recently observed breakup of global coherent oscillations [2] reflected here in declining spatial slow-wave coherence (Figure 3.B.3) – a finding that we confirmed also in the human EEG data (Figure 1.C.1). We used a simple negative feedback on firing activity to implement homeostatic plasticity. Specifically, the model predicted that an acute increase in the firing rate results in a faster homeostatic downscaling of synapses. Thus, we predicted a reduced decay of slow-wave decay (in power and coherence) in the hours after stimulation (Figure 3.B.2–B.3). Human SWO subsequent to stimulation were indeed modulated as predicted (Figure 1.B.3–C.3). The results are further confirmed by the precise agreement of model predictions with the varying effect size observed across electrodes (Figure 4.B–4.C).

The choice of a target firing rate was made to reproduce the experimentally observed decrease in firing rate during slow-wave sleep as reported in *in-vivo* experiments [11]. Previous models of SWO implemented a reduction of synaptic strength explicitly [9] or implicitly using STDP [64]. More complex models of plasticity, such as the BCM model [65] are expected to lead to similar predictions.

An alternative interpretation of the observed reduction in decay rate after stimulation may be an alteration of sleep stages, e.g. the first slow waves stage was disrupted. However, it is not clear how this hypothesis would lead to different effects at different electrode locations. It is also possible that fields have a direct effect on synaptic strength, but current literature suggests that very specific conditions need to be satisfied for plastic effects to be observed. While we made no direct observation of firing rates nor synaptic strengths, the agreement between the present multi-scale model and the human EEG data does support the hypothesis that field-induced cell polarization results in an increase of firing rate and that this accelerates synaptic downscaling during oscillatory transcranial stimulation.

## Materials and Methods

### Human EEG data after stimulation in sleep

EEG data was recorded on human subjects from the beginning of the night sleep until wake the next morning in the study described by [14]. Briefly, transcranial stimulation with slow-oscillating currents (ON/OFF at 0.75 Hz with trapezoid waveform) was performed after subjects had attained stable stage 2 or deeper non-rapid eye movement sleep (according to [66]). Stimulation was repeated altogether 5 times for 5 minutes followed by 1 minute intervals without stimulation (total of 25 minutes stimulation plus four one-minute intervals). Anodal stimulating electrodes were placed bilaterally at F3 and F4 and cathodal electrodes on mastoids M1 and M2 (10/20 system, Figure 1.A.1). Current intensity on each hemisphere oscillated between 0.26 mA (on) and 0.0 mA (off) and was below perception. To assure that stimulation intensities were below perception thresholds we stimulated subjects for 10 seconds (active and sham) when subjects where in bed but lights were still on. Immediately after, subjects were asked whether they had felt anything on their head. The subjects responses did not differ between the active stimulation or sham stimulation, indicating that the stimulation was indeed below perception. Note that the stimulation used in the study are significantly lower than the maximum used during transcranial stimulation (2 mA, [13], [55]) and so well below the current amplitudes considered safe for human studies [67], [68]. To test further for possible side effects, heart rate was monitored during sleep, i.e. during stimulation and thereafter. No obvious changes in heart rate were observed during the stimulation. The experimental protocol was approved by the ethics committee of the University of Lübeck.

For the present analysis EEG data with complete sleep scores included 10 subjects for the sham conditions and 13 subjects with active stimulation. Paired tests were thus limited to 10 subjects. Acute entrainment of EEG to the oscillatory stimulation on this data has been previously reported [14]. However, this previous analysis did not consider the phase of entrainment nor slow-wave spatial coherence, and more importantly, it did not analyze long term decay of SWO in the hours following stimulation.

#### Power and spatial coherence changes in the human EEG data

Slow-wave power varies significantly with different sleep stages. In order to compare slow-wave power from different recording sessions it is therefore important to align sleep stages. The EEG data were aligned to the first uninterrupted 1 minute period in sleep stage 2. With this, the SWO power (0.5–1 Hz) in the minute preceding the stimulation period did not differ between sham and stimulation conditions (N = 10 and N = 13 for sham and stimulation conditions respectively, 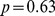, two-sample Kolmogorov-Smirnov test). SWO power was measured for each electrode in periods of 40 seconds by averaging power in the corresponding frequency bins after Fourier transform. Spatial coherence was determined from the normalized cross-correlation by Fourier transforming, squaring, and averaging across SWO frequency bins. Values are computed for each electrode by averaging coherence of all pairs involving the electrode. These power and coherence measures are obtained for all 40 seconds intervals. Their decay rate during the night was measured as the slope of these curves using a linear robust fit. The fit considered a 4.5 hour period starting at the end of the stimulation until 30 min before the end of the shortest signal (to avoid contamination from awakening). Non-parametric statistics were obtained by randomizing the labels (sham vs stimulation) and computing mean decay rates with random labels. p-values were computed using these shuffle statistic. Correction for multiple comparisons across electrodes controlled the false-discovery rate (FDR).

### Computational model

#### Single-cell model

We restrict our model to a single compartment neuron. This simplification omits the effects of fields on the dendritic arbors [47] yet is sufficient to describe effects on spiking activity [19], [48]. We used Izhikevich's model [69], [70] with a set of parameters that reproduces the physiological spiking behaviors of cortical neurons. The equations describing the neuronal dynamics and the details on the network model can be found in [69] and in our previous study [19].

#### Network model

The network model consists of 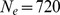 excitatory neurons and 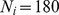 inhibitory neurons arranged at random on a 2D lattice. When a spike is elicited by neuron 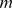 the synaptic input current to neuron 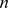 is given by the synaptic currents of AMPA and NMDA channels (for excitatory pre-synaptic neurons) and 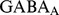, 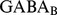 channels (for inhibitory pre-synaptic neurons). The synaptic conductances are described by a first-order linear kinetics 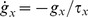 (where x = AMPA, NMDA, 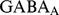, 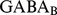) with 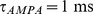, 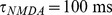, 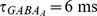, 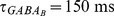. When a pre-synaptic neuron fires an action potential, the synaptic conductance of the post-synaptic neuron increases in average by 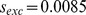 or 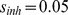 for excitatory or inhibitory connections respectively. The synaptic currents are then [70]:
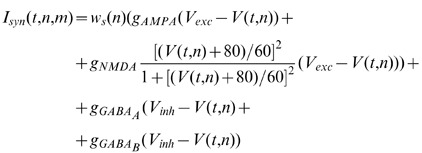
(1)where 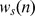 represents a modulatory homeostatic factor (see below), the conductances are 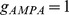, 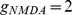, 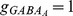 and 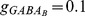, 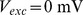, 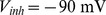 are the reversal potentials for excitatory and inhibitory synapses respectively.

Neuron receive excitatory input from a 5×5 neighborhood and inhibitory input from a 3×3 neighborhood with periodic boundary conditions. In any simulation run, parameters of the Izhikevich model as well as synaptic strength 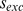 and 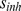 were chosen at random following a normal distribution with standard deviation equal to 5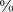 of the average value.

#### Model for the generation of slow-wave oscillations

At the network level, slow waves oscillations are thought to reflect a periodic transition between an active “UP” state and a quiescent “DOWN” state. To simulate elevated firing activity of the UP state we increased the level of intrinsic excitability of neurons by increasing the variable 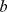 in Izhikevich's voltage equation [69]. If firing rate in such an active UP state is very high then a variety of factors may contribute to a gradual decay of neuronal excitability. Thus, we made the dynamics of this variable 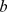 activity-dependent to reflect a negative feedback. Specifically, in our model the instantaneous firing rate of a neuron modulates the excitability of that same neuron as follows:
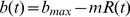
(2)where 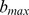 is the value of the parameter 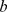 in steady state conditions (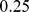 and 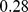 for excitatory and inhibitory neurons respectively); 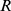 reflects the neuron's firing rate (low-pass filtered spike train with time constant 0.9 s) and 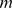 is a proportionality constant (set in the simulations to 6). Physiologically, such a negative feedback on excitability with this time scale has been variably ascribed to neuromodulators (acetylcholine, norepinephrine), ionic concentrations (potassium and calcium), ionic channels (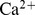-dependent potassium channels, persistent sodium channels) or metabolic support.

#### UP/DOWN states can result from activity-dependent slow recovery dynamics in a balanced excitatory/inhibitory network

**Figure 5:**
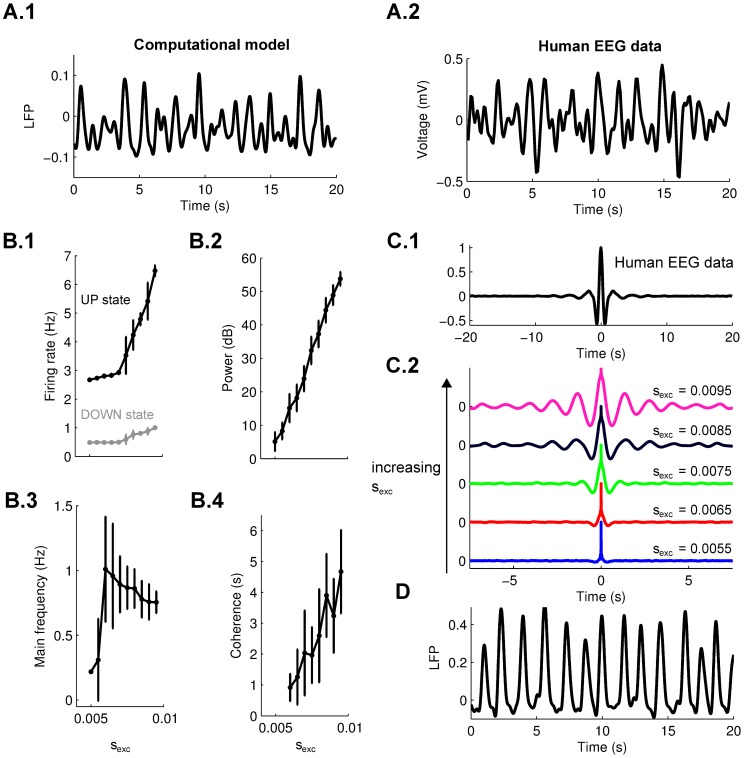
Slow waves features in the computational model. **A.1**: Simulated local field potentials (LFP) in the computational model (low-pass filtered, cutoff frequency 2.5 Hz). **A.2**: Human EEG signal during slow-wave sleep (low-pass filtered, cutoff frequency 2.5 Hz). **B**: Dependence on excitatory connections strength of firing rate during the UP and DOWN states (**B.1**), power (**B.2**), main frequency (**B.3**) and coherence (**B.4**) of slow-wave oscillations (n = 5 simulations per data point, error bars indicate standard deviation). **C.1**: Example of auto-correlation of slow waves in the human EEG experiments (average of 5 subjects). **C.2**: Auto-correlation of simulated slow waves increasing the strength of excitatory connections. **D**: Example of simulated slow waves oscillations in the case of high synaptic connection strength (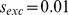).

The negative feedback on excitability down-regulates excitability so that the active UP state is eventually exhausted and comes to an end. The network thus enters a quiescent state with little, if any activity. This DOWN state persists until 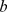 recovers, at which point any small perturbation can jump-start the UP-state, propagating like an avalanche through the network [71]. This network model reproduced the regular UP and DOWN states transitions typical of slow-wave oscillations (Figures 2.B.2). In the network model we take the post-synaptic currents averaged across all neurons as a measure of local-field potentials (LFP) – since physiological LFPs are thought to reflect synaptic activity. With the present parameter settings the frequency and bandwidth of the network LFP was in the range of 0.5–1 Hz (Figure 5.A.1). This is the dominant band of slow-wave activity (0.5–4 Hz) in the human EEG (Figures 5.A.2) and is referred to as slow-wave oscillation [38]. For Figures 3 and 4 the LFP was estimated in four subregions of the network (in arrays of 11×11 neurons) and each LFP treated analogously to the multiple electrodes in the EEG. From these LFPs power and spatial coherence were calculated in the same way than the EEG data.

#### Power and coherence of slow-wave oscillations depend on synaptic strength

In the model the firing rate during the UP states and the power of slow-waves depend strongly on the strength of excitatory connections, 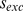 (Figures 5.B.1–5.B.2). The configuration of parameters chosen here simulated UP states with an average firing rate of 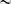
5 Hz, compatible with slice experiments (2–10 Hz, [27]). Stronger excitatory connections would produce higher firing rate and stronger power of slow-waves, but the parameters where chosen to replicate the irregular EEG rhythms, as seen in Figure 5.A.2. In particular, while the frequency of the oscillations does not depend strongly on the range of excitatory connections (in the 0.5–1 Hz range, Figure 5.B.3), a critical characteristic of slow-wave oscillations in human EEG data is the short coherence time (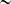
3 cycles, measured from the EEG data, Figure 5.C.1). The strength of excitatory connections (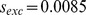) was chosen to reproduce the short coherence time of EEG data (Figure 5.B.4–5.C.2). Increasing the strength of excitatory connections allows to reproduce the strongly regular pattern typical of slow-wave activity induced in brain slices (Figure 5.D).

#### Model of effect of electric field

Most somata of inhibitory neurons remain largely unaffected by extracellular fields due to their symmetric location between dentritic arbors [31]. In contrast, somata of asymmetric pyramidal cells are incrementally polarized by uniform extracellular fields proportionally to the applied field magnitude 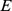 [47], [48]:
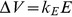
(3)where 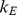 is the sensitivity of the membrane to the field and depends on cell geometry and field orientation. We simulated here the effects of the field as a current injection to each excitatory neuron. This approach have been already successful in describing the effects of weak fields on gamma activity in rat hippocampal slices [19] and on slow waves in ferret cortical slices [28]. A capacitive term in Izhikevich's model converts this current input into a low-pass filtered membrane voltage response. Specifically, a current 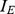 results in a steady-state incremental polarization 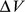 above the resting membrane potential. With the present parameters the relationship between injected current and induced polarization was measured as 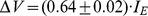 where 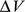 is in mV. We assume that a 1 V/m electric field can polarize the soma by 0.2 mV (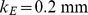, typical value for rat hippocampal pyramidal cells). With this we can estimate the relationship between electric field and applied current as 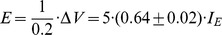. All figures use this conversion term when displaying values of electric field.

The total input current 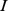 to the 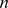-th neuron is then given by:
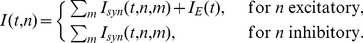
(4)


#### Model for homeostatic plasticity

There are different known types of homeostatic plasticity, involving different possible mechanisms [10]. The plasticity considered here affects the excitatory synaptic connections based on the firing rate of the post-synaptic neuron 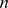 [72],
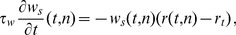
(5)where 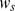 is a factor that modulates excitatory synapses only, 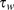 is the time constant of this long-term process (minutes), 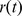 is the instantaneous firing rate of the post-synaptic neurons computed as the inverse of the inter-spike interval (ISI) and 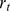 is the target firing rate. This homeostatic rule states that inputs to a post-synaptic neuron that is spiking faster than the target firing rate become weaker, while inputs to neurons not firing enough become stronger. The values of the constant were chosen as 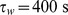 and 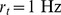. These values were chosen to reproduce changes of SWO power comparable with those measured during the night in the human EEG experiments.

### Finite Element Model of transcranial electrical stimulation

**Figure 6:**
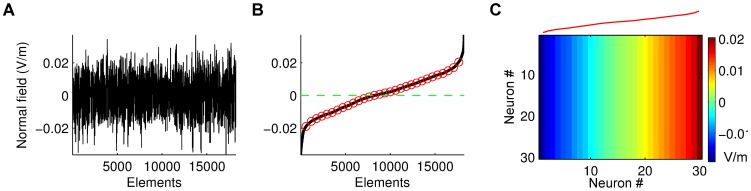
Workflow to use the FEM analysis with the computational model. **A**: Example of distribution of the normal component of the electric field under the electrodes considered in the FEM analysis (in this case Pz electrode). **B**: Radial field magnitudes in **A** were sorted and sampled in 30 location (3.12 percentile extremes were excluded). **C**: The sampled electric fields are then used for each column of neurons in the 2D lattice of the network model.

The FEM computations follow a previous study [18]. Briefly, an anatomical MRI with 1 mm resolutions for an adult male was segmented and different tissues (gray matter, white matter, cerebrospinal fluid, skull, scalp, eye region, muscle, air, and blood vessels) were assigned conductivity values from the literature. Virtual electrodes were placed as in the human experiment and a finite-element mesh was generated. To compute electric field distribution in the brain the Laplace equations with Neumann boundaries were solved in COMSOL Multiphysics 4.2 (Burlington, MA) with electrodes drawing 0.26 mA. The radial component of the resultant electric field was computed as the dot product of field vectors with a unit vector that is normal to the cortical surface. These radial components were collected in a volume of a 35 mm diameter around each EEG electrode (Figure 6.A shows radial fields at mesh points of the FEM within such a volume). These values were then sorted (Figure 6.B) and the resulting field profile was applied along one direction of the 2D network lattice (Figure 6.C). The top and bottom 3.12 percentile were exclude and amplitudes scaled to an average of 0.93 V/m.

**Figure 7:**
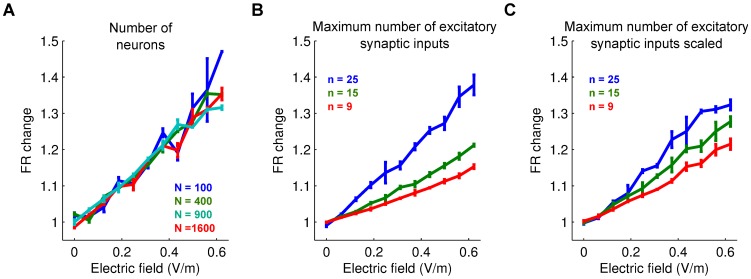
The effects of electric fields on firing rate depend on synaptic connectivity. Normalized change in the average neuronal firing rate as a function of the number of neurons in the network model (**A**) or the numbers of pre-synaptic excitatory inputs (**B**). **C**: Same than in **B** but with constant total synaptic input. Effect of fields on firing rate depends on number of input synapses and not network size.

The fields computed by the FEM are significantly smaller than what we used in the network simulations. However, there are a number of parameters that may magnify the specific effect size. The polarization of the cell membrane in response to applied fields used here was based on in-vitro experiments in rat [48]. Human cortical cells are larger, which may result in larger membrane polarizations [31]. More importantly, we observed for the present model that the effect of polarization on network firing rate is an increasing function of the number of incoming synaptic connections (Figure 7). A realistic network architecture with hundreds if not thousands synaptic inputs is thus expected to lead to a larger effect size.

## Supporting Information

Figure S1 Example traces of the analysis performed on the EEG data. The decay of slow-wave oscillations was estimated by fitting (in a log-scale) power and spatial coherence after the stimulation (see Materials and Methods). **A–B**: Decay of the power of slow-wave oscillations during the night (Fz electrode, green: sham condition, red: stimulation condition) for two representative subjects.
(TIFF)

Figure S2 Entrainment of slow oscillatory activity by applying weak electrical stimulation. **A**: Coherence (mean vector strength, maximum = 1) between model LFP and applied slow-oscillating field as a function of field intensity and fractions of neuron polarized in either direction. **B.1**: Relative change of the duration of the DOWN state in the case of cathodal (blue) or anodal (red) stimulation (0.31 V/m). **B.2**: Relative change of the duration of the UP state in the case of cathodal (blue) or anodal (red) stimulation (0.31 V/m). **C.1**: Entrainment of slow-wave oscillations immediately after the stimulation in the human EEG data (shown here for Pz electrode). The dark gray bar indicate the 10 s interval (delimited by the dashed magenta line) where the distribution of phases of the oscillations across trials and subjects is significantly different from being uniform. The same analysis performed on the following 10 s does not produce results statistically different from a uniform distribution (no preferential phase). **C.2**: Distribution of phases relative to figure **C.1** considering all the trials and all the subjects. The 5 stimulation periods for all the subjects were aligned and the exponential decay from the AC-coupled amplifier was removed. The residual was fit to as sinusoid in frequency, phase and amplitude. Entrainment phase was only analyzed for the Pz electrode as this was the electrode with the smallest stimulation artifact. Note that the EEG recording equipment was AC-coupled resulting in a constant phase delay. Thus absolute value of phase is not relevant here. Nevertheless, a consistent phase across subjects despite anatomical differences is indicative of the predicted entrainment to a preferred phase.
(TIFF)
