# Supplementary figures and images for: Transcranial Electrical Stimulation Accelerates Human Sleep Homeostasis
Source: PLoS Comput Biol. 2013 Feb 14;9(2):e1002898. doi: 10.1371/journal.pcbi.1002898 (PMC3573006; doi:10.1371/journal.pcbi.1002898)

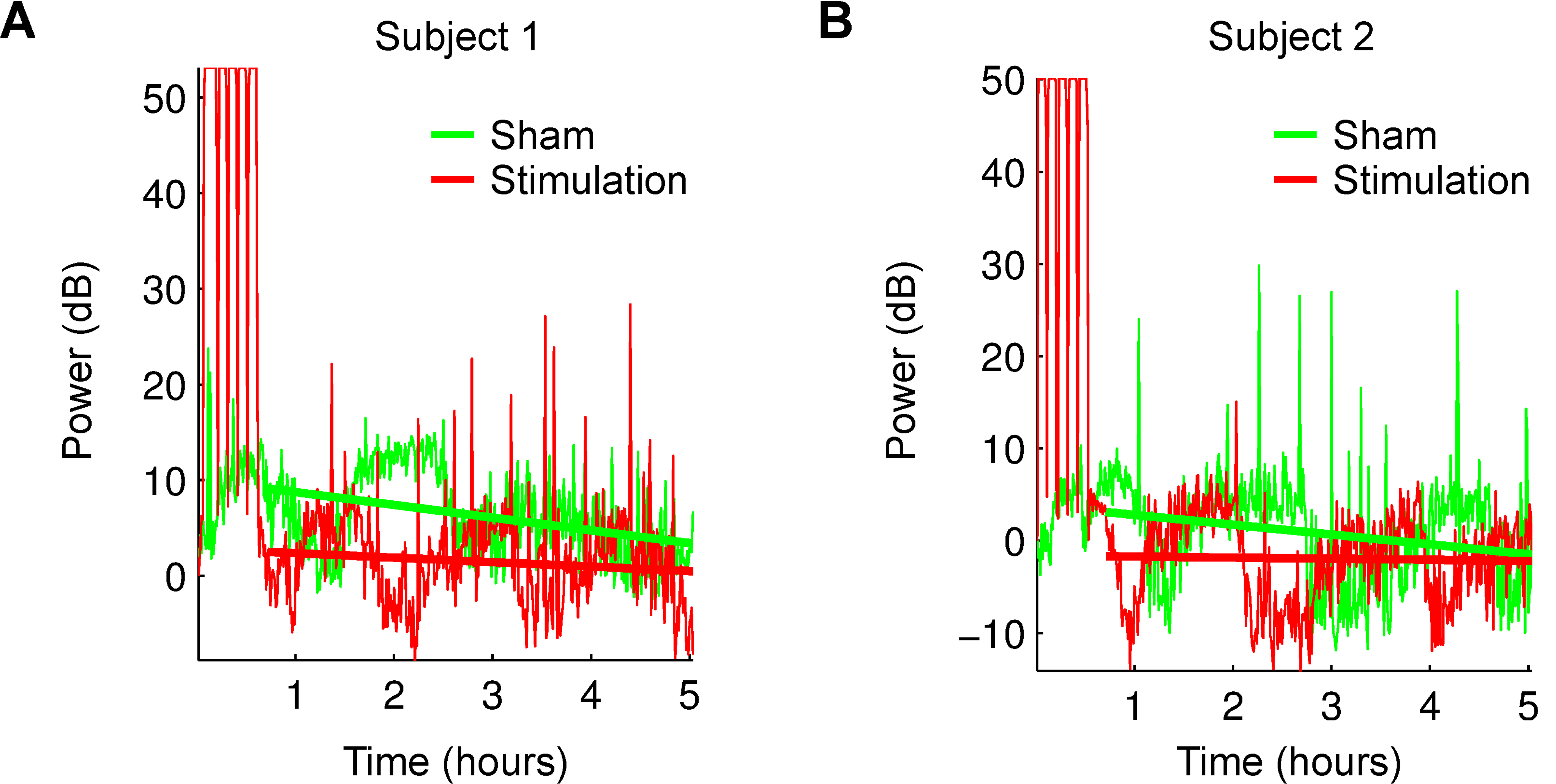

Supplement: Figure S1 — Example traces of the analysis performed on the EEG data. The decay of slow-wave oscillations was estimated by fitting (in a log-scale) power and spatial coherence after the stimulation (see Materials and Methods). A–B: Decay of the power of slow-wave oscillations during the night (Fz electrode, green: sham condition, red: stimulation condition) for two representative subjects. (TIFF) [file pcbi.1002898.s001.tiff]

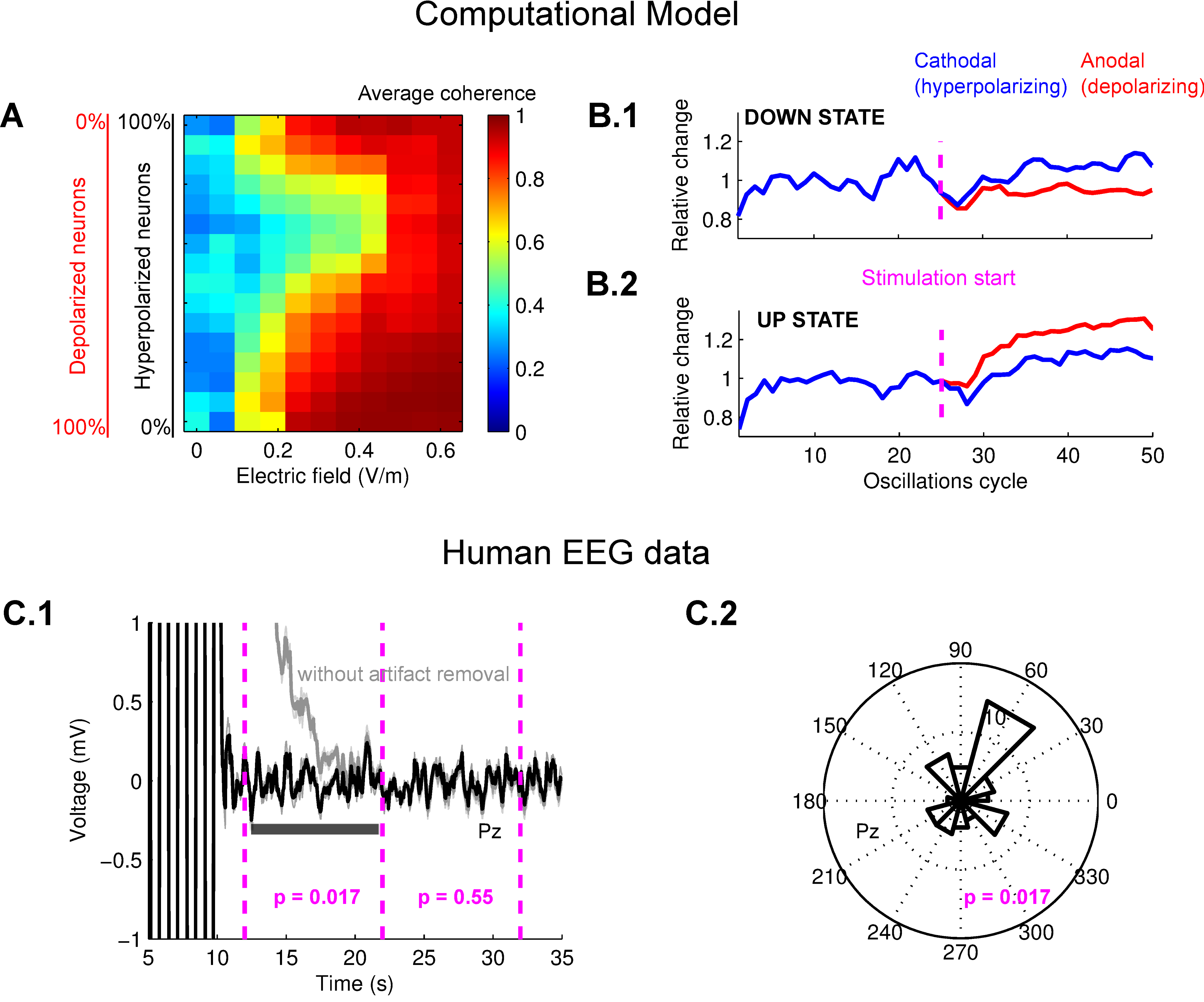

Supplement: Figure S2 — Entrainment of slow oscillatory activity by applying weak electrical stimulation. A: Coherence (mean vector strength, maximum = 1) between model LFP and applied slow-oscillating field as a function of field intensity and fractions of neuron polarized in either direction. B.1: Relative change of the duration of the DOWN state in the case of cathodal (blue) or anodal (red) stimulation (0.31 V/m). B.2: Relative change of the duration of the UP state in the case of cathodal (blue) or anodal (red) stimulation (0.31 V/m). C.1: Entrainment of slow-wave oscillations immediately after the stimulation in the human EEG data (shown here for Pz electrode). The dark gray bar indicate the 10 s interval (delimited by the dashed magenta line) where the distribution of phases of the oscillations across trials and subjects is significantly different from being uniform. The same analysis performed on the following 10 s does not produce results statistically different from a uniform distribution (no preferential phase). C.2: Distribution of phases relative to figure C.1 considering all the trials and all the subjects. The 5 stimulation periods for all the subjects were aligned and the exponential decay from the AC-coupled amplifier was removed. The residual was fit to as sinusoid in frequency, phase and amplitude. Entrainment phase was only analyzed for the Pz electrode as this was the electrode with the smallest stimulation artifact. Note that the EEG recording equipment was AC-coupled resulting in a constant phase delay. Thus absolute value of phase is not relevant here. Nevertheless, a consistent phase across subjects despite anatomical differences is indicative of the predicted entrainment to a preferred phase. (TIFF) [file pcbi.1002898.s002.tiff]
